# Salt Taste, Nutrition, and Health

**DOI:** 10.3390/nu12051537

**Published:** 2020-05-25

**Authors:** Albertino Bigiani

**Affiliations:** Dipartimento di Scienze Biomediche, Metaboliche e Neuroscienze, Università di Modena e Reggio Emilia, 41125 Modena, Italy; albertino.bigiani@unimore.it; Tel.: +39-059-205-5349

The sodium ion (Na^+^) is essential for life. Na^+^ is the main cation in the extracellular fluid bathing all our cells, and it is also a key element in many body secretions. In addition to determining the extracellular fluid osmolality, Na^+^ is involved in several physiological processes that would be impaired by its deficiency—without Na^+^, neurons and muscle cells would not be able to generate electrical impulses, the intestinal adsorption of nutrients would be undermined, and the kidneys would not work properly. To maintain stable levels of Na^+^ in our body, losses through kidneys, the gastrointestinal tract, and sweating have to be balanced by the ingestion of foods containing this mineral. Our ability to recognize Na^+^ relies mainly on the activity of the taste system. Usually, we consume sodium in the form of sodium chloride (NaCl), the common table salt. Moderate concentrations of salt induce a specific “salty” sensation that is appealing and appetitive for us. However, saltiness can be perceived as pleasant or unpleasant according to the salt concentration and the medium in which salt occurs. For example, we dislike salt concentrations >150 mM in aqueous solution, but we found them palatable when salt is in foodstuffs. The positive hedonic tone (liking) of salt taste may expose us to salt overconsumption. This is further exacerbated by the widespread use of NaCl as preservative and as flavoring agent in processed foods. When consumed in excess, salt is detrimental to health: in fact, excessive sodium intake is linked to the development of hypertension and related pathologies.

This Special Issue provides a contribution to our understanding of salt taste and of its impact on nutrition and health. Although Na^+^ is a simple chemical, the biological processes underlying its handling in our body are quite complex. Figure 1 shows a simplified scheme of sodium balance mechanisms. In the following paragraphs, I will discuss some of the new findings with relation to the processes highlighted in Figure 1: Na^+^ detection, eating behavior, blood pressure regulation, and Na^+^ output regulation.

## 1. Na^+^ Detection

**Figure 1:**
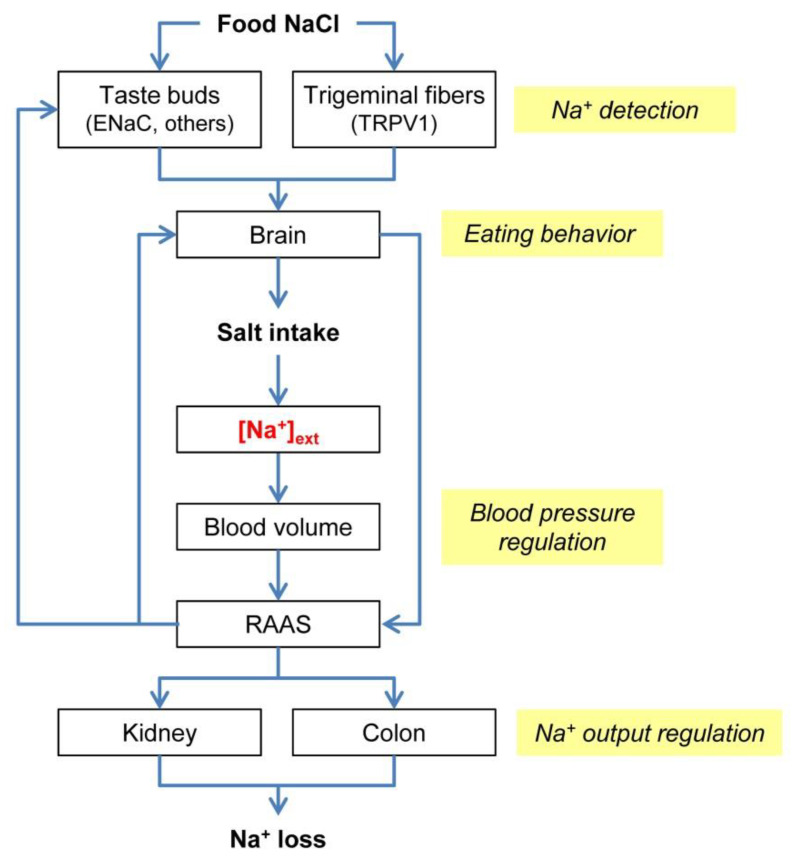
**Sodium balance**. An adequate extracellular concentration of Na^+^ (*[Na^+^]_ext_*) is vital for the functioning of our cells. Diet provides the daily amount of Na^+^ to balance off losses through kidney, intestine, and sweating (not shown). Food Na^+^ is detected mainly by taste buds, which contained sensory cells able to sense this cation through sodium receptors (*ENaC* and others not yet identified). Taste information is fundamental for recognizing the quality and the intensity of saltiness. However, large salt concentration may also activate trigeminal nerve endings, which contain another “salt” receptor (*TRPV1*). This sensory pathway is believed to provide information on stimulus intensity in supra-threshold salt concentration range. Chemosensory signals are processed in the brain for conscious perception and for sodium appetite regulation. The outcome of central processing guides salt intake (Na^+^ input). Note that oral chemosensory input provides feed-forward signals alerting central neurons on the presence of sodium-containing foods before Na^+^ absorption has occurred in the gut. Once absorbed, ingested Na^+^ is confined mainly to the extracellular space, and changes in its extracellular concentration affect blood volume and blood pressure, which in turn influence the renin-angiotensin-aldosterone system (*RAAS*). RAAS controls the amount of Na^+^ lost by kidney and colon (Na^+^ output), and also provides feedback to neural centers and taste buds to regulate sodium appetite and salt sensitivity, respectively. In turn, the brain modulates renin secretion through baroreceptor reflexes. Papers in this Special Issue touch upon some of the processes (Na^+^ detection, eating behavior, blood pressure regulation, Na^+^ output regulation) that are associated with the handling of food Na^+^ by our body. Note that, for simplicity, other factors involved in sodium balance, such as the atrial natriuretic peptide, are not shown.

The principal detectors of food Na^+^ are the taste cells, specialized epithelial cells clustered in the taste buds of the oral mucosa. Na^+^ activates these sensory cells by interacting with specific membrane proteins (sodium receptors) located in their apical portion, where they reach contact with the saliva. Taste cells then relay sensory information to nerve fibers, which transmit electrical impulses to the brain (Figure 1). The use of the diuretic drug, amiloride, has allowed researchers to distinguish two parallel signaling routes in most mammals: the amiloride-sensitive (AS) pathway and the amiloride-insensitive (AI) pathway. The epithelial sodium channel (ENaC), which is blocked by amiloride, works as a sodium receptor in the AS pathway. Sodium detection by the AI pathway is not affected by amiloride. The AS mechanism is highly specific for Na^+^ detection, whereas the AI one is more broadly tuned to detect also other cations. However, the identity of the AI salt receptor(s) is(are) still unknown. These gustatory pathways transmit information on the stimulus quality (saltiness) and its intensity (salt concentration). Four papers provide data on the mechanisms underlying Na^+^ detection by taste cells. Bigiani [1] reviews our current understanding on the possible involvement of ENaC in the initial events of Na^+^ detection in humans. Although this oligomeric protein works as low-salt receptor in laboratory mammals, available data “seem to favor a role for ENaC downstream of the initial receptive events” in human salt taste. Shigemura et al. [2] provide compelling evidence that some components of the renin-angiotensin-aldosterone system (RAAS) occur in mouse taste buds. Circulating RAAS plays a key role in the regulation of sodium balance by controlling sodium excretion and by providing feedback to specific neural circuits and taste buds to regulate sodium appetite and salt sensitivity, respectively (Figure 1). The existence of components of RAAS in taste buds suggest that the activity of salt-sensitive cells could be also modulated locally in response to “random perturbations in the oral cavity during feeding and drinking”, and this could affect information sent to central neurons. The authors suggest that local RAAS could be involved in “short-term feedforward regulation predicting changes in body fluid composition”. Cattaneo et al. [3] found that the abundance of certain bacterial taxa on the tongue dorsum of healthy volunteers was negatively correlated with salt taste sensitivity (assessed by recognition threshold for NaCl solutions): namely, the more abundant those taxa, the lower the salt taste threshold. This result underscores the importance of the so-called perireceptor events in salt detection and possibly in defining the inter-individual differences in salt taste perception. The authors suggest that “oral microbiota composition deserves to be considered as an influencing variable when investigating perireceptor events involved in chemosensory processes”. Lossow et al. [4] studied the effect of changes in dietary salt content on mRNA expression for ENaC subunits in mouse taste buds. They found that mRNA levels were not affected either by low-salt or by high-salt diet. Thus, taste function does not seem to play a major role in body adjustment to sodium imbalance, at least on the basis of the mRNA expression levels for the sodium receptor in mice.

In addition to taste cells, food Na^+^ can be detected by trigeminal nerve endings, which are widely distributed throughout the lingual mucosa and also around taste buds. This sensory pathway is activated by high salt concentrations and likely provides information on salt concentration in supra-threshold range and also to avoid ingestion of hyperosmotic salt that could impair extracellular osmolality. Trigeminal nerve endings express the transient receptor potential vanilloid 1 receptor (TRPV1), an ion channel proposed to work as “salt” receptor. Rhyu et al. [5] provide interesting data on the involvement of trigeminal pathway in salt detection. These authors found that some “kokumi” peptide fractions isolated from Ganjang, a typical Korean soy sauce, were able to increase the perceived salt taste intensity in human volunteers, that is, they worked as a salt taste enhancer. Kokumi refers to tasteless compounds able to improve persistency and mouthfulness (mouth-filling sensation) as well as to enhance some of basic taste qualities, including salty taste, as demonstrated here. By recording the activity of chorda tympani (CT) taste nerve in rats, Rhyu et al. [5] found that these taste active peptides did not affect the ENaC-mediated AS pathway, but the AI one. However, the enhancing effect was likely due an interaction between trigeminal nerve endings containing TRPV1 and taste cells of the AI pathway. These results suggest “a novel relationship between trigeminal system and salt taste perception”.

## 2. Eating Behavior

Eating behavior is a complex activity that has evolved to assure the proper ingestion of chemicals for body metabolism and homeostasis. In humans, the consumption of salty foods is driven not only by the integrated neuroendocrine mechanisms regulating the activity of central appetite neurons (Figure 1), but also by the combination of several other factors, including genetic context and non-homeostatic influences, such as learning, cultural factors, and personal habits. All these factors significantly affect the preference for salty foods, and therefore salt intake, determining individual variability. Three papers address human eating behavior in terms of possible association between salt taste perception and salt preference or dietary habits. Cattaneo et al. [3] found a correlation between decreased salt taste sensitivity and consumption of bakery and salty baked products in healthy, normal-weight, 18-30-year old volunteers. These findings indicate that inter-individual variation in salt perception may affect habitual salt consumption. As reported above, they also found a correlation between relative abundance of certain bacterial taxa and salt taste sensitivity. Thus, oral microbiota may influence food preference. The possible association between salt taste genotype and eating behavior has been studied by Ferraris et al. [6] in a large-sized (*n* = 536) and well-characterized elderly cohort in Australia. They evaluated the association between the occurrence of single nucleotide polymorphisms (SNPs) for the TRPV1-encoding gene and individual salt intake. TRPV1 is a salt receptor expressed in trigeminal nerve endings, which may contribute to the perception of NaCl, especially when salt concentration is high. Their findings indicate that there is no association between TRPV1 SNP and salt intake in the analyzed elderly cohort, suggesting that, at least for people aged 65 years or older, the TRPV1 genotype is not crucial in defining salt consumption. Veček et al. [7] performed a cross-sectional study on general population of Dalmatia, Croatia (*n* = 2798 subjects) to determine possible association between salt taste perception, Mediterranean diet, and Metabolic Syndrome (MetS). They found that there were no differences in the overall Mediterranean diet compliance between subjects with different salt taste threshold. However, they found that “subjects with higher salt threshold added salt to their food more frequently compared to subjects with both lower and intermediate threshold”. This means that individuals with higher salt sensitivity (lower taste threshold) could have a reduced salt intake. Interestingly, they found that these subjects also showed lower prevalence of MetS.

## 3. Blood Pressure Regulation

Nervous and endocrine mechanisms assure a proper level of hydraulic pressure in the large arteries to sustain an adequate blood perfusion throughout the body. Salt intake may affect blood pressure. Ingested Na^+^ is confined into the extracellular compartment. Here, through water retention, Na^+^ sets the overall volume, including the blood volume. In turn, this affects blood pressure (Figure 1). It is therefore clear that one of the consequences of an increased salt intake is likely an increase in blood pressure. Several studies underscore the role of excessive salt consumption in the development of hypertension, which represents a component of MetS. The association between salt intake and blood pressure is addressed in this Special Issue. Ferraris et al. [6] found (see above) that SNP for the gene encoding the salt receptor, TRPV1, was not associated with a variation in salt intake in an elderly cohort. Consistently, neither systolic nor diastolic blood pressure varied by genotype. The authors acknowledge that “as the cohort was 65 years and older, the results are not necessarily generalizable to the wider adult or youth population” since aging may affect genetic expression. In their cross-sectional study on the general population of Dalmatia (Croatia), Veček et al. [7] found that “subjects with higher salt taste threshold were on average older than those with lower threshold”. This means that the ability to recognize salty stimuli blunts with age. In addition, they found that “age was also negatively correlated with salt taste intensity perception”, evaluated with supra-threshold testing. As a whole, these results suggest that aging affects salt taste performance. Veček et al. [7] also found that high blood pressure, a MetS component, was more common among subjects with higher salt recognition threshold, that is, those with lower salt sensitivity. Mun et al. [8] studied the effect of Doenjang, a traditional Korean seasoning with a high salt content obtained by soybean fermentation, on the blood pressure in rats, which were fed a high-salt diet with or without Doenjang. Interestingly, blood pressure was significantly lower in the first group, although the administered salt content was similar in the two groups. In addition, RAAS was also affected: renin and aldosterone levels were decreased in mice fed with Doenjang. Likely, other chemicals occurring in Doenjang as well as its microbial community offset the effect of salt intake on blood pressure. Thus, their results suggest that “eating traditional salty fermented food is not a direct cause of hypertension, and the intake of Doenjang in normal healthy animals improved blood pressure”. In line with the goal of reducing salt consumption without affecting the palatability of foodstuffs, Rhyu et al. [5] identified kokumi active peptides that could be used as salt taste enhancers. Clearly, this would be beneficial for controlling blood pressure, because less salt would be necessary to have the same saltiness perception when these peptides are present in foods.

## 4. Na^+^ Output Regulation

Renal excretion and losses through the gastrointestinal tract represent the output of the homeostatic system controlling the extracellular concentration of Na^+^ ([Na^+^]_ext_). Variations in salt intake lead to changes in [Na^+^]_ext_, which in turn affect, through expansion/reduction of extracellular volume to correct osmolality, blood volume and RAAS (Figure 1). RAAS targets the renal nephron and the colon, where aldosterone affects sodium reabsorption through ENaC to match changes in sodium intake. Lossow et al. [4] found that changes in dietary salt content in mice affected ENaC mRNA expression levels in kidney and distal colon. As reported above, they also analyzed ENaC expression in taste buds, since in this mammal ENaC works as sodium taste receptor. They data clearly indicate that “colon and kidney seem to be of greater importance to compensate imbalanced sodium homeostasis than gustatory tissue based on the monitored ENaC expression levels”. These results underscore the importance of an adequate regulation of sodium output to compensate changes in [Na^+^]_ext_ due to variations in salt intake. In this regard, it is worth noting that salt taste works as a feedforward mechanism that can reduce sodium appetite to prevent overconsumption. On the contrary, Na^+^ output is the expression of a homeostatic regulation, which uses compensatory feedback mechanisms to stabilize [Na^+^]_ext_ (Figure 1).

## 5. Final Note

I would like to thank all the authors in this Special Issue for providing their new research data on salt taste mechanisms and on the role of salt taste in nutrition and health. I am sure their valuable contributions will be appreciated by the readership of *Nutrients*, as well as by the scientific community.
